# Frailty, Pitavastatin, and Major Adverse Cardiovascular Events Among People With HIV

**DOI:** 10.1016/j.jacadv.2025.102077

**Published:** 2025-08-20

**Authors:** Kristine M. Erlandson, Ariela R. Orkaby, Triin Umbleja, Heather J. Ribaudo, Todd T. Brown, Markella V. Zanni, Marissa R. Diggs, Sarah M. Chu, Kathleen V. Fitch, Carl J. Fichtenbaum, Carlos Malvestutto, Judith A. Aberg, Gerald S. Bloomfield, Judith S. Currier, Karen T. Tashima, Marcus Vinícius Guimarães de Lacerda, Sonya L. Heath, Michael T. Lu, Alan Landay, George A. Kuchel, Pamela S. Douglas, Steven K. Grinspoon

**Affiliations:** a Department of Medicine, University of Colorado Denver- Anschutz Medical Campus, Aurora, Colorado, USA; b New England GRECC (Geriatric Research, Education, and Clinical Center), VA Boston Healthcare System, Boston, Massachusetts, USA; c Division of Aging, Department of Medicine, Brigham & Women’s Hospital and Harvard Medical School, Boston, Massachusetts, USA; d Department of Biostatistics, Harvard TH Chan School of Public Health, Boston, Massachusetts, USA; e Department of Medicine, Johns Hopkins Bloomberg School of Public Health, Baltimore, Maryland, USA; f Metabolism Unit, Massachusetts General Hospital and Harvard Medical School, Boston, Massachusetts, USA; g Department of Medicine, University of Cincinnati College of Medicine, Cincinnati, Ohio, USA; h Department of Medicine, Ohio State University Medical Center, Columbus, Ohio, USA; i Department of Medicine, Icahn School of Medicine at Mount Sinai, New York, New York, USA; j Duke Clinical Research Institute, Duke Global Health Institute and Department of Medicine, Duke University, Durham, North Carolina, USA; k Department of Medicine, University of California-Los Angeles, Los Angeles, California, USA; l Department of Medicine, Warren Alpert Medical School of Brown University, Providence, Rhode Island, USA; m Fundação de Medicina Tropical Dr Heitor Vieira Dourado and Fiocruz, Manaus, Brazil; n Department of Medicine, University of Alabama Birmingham School of Medicine, Birmingham, Alabama, USA; o Cardiovascular Imaging Research Center, Department of Radiology, Massachusetts General Hospital and Harvard Medical School, Boston, Massachusetts, USA; p Department of Medicine, University of Texas Galveston Branch, Galveston, Texas, USA; q Department of Medicine, University of Connecticut, Farmington, Connecticut, USA; r Duke Clinical Research Institute, Duke University School of Medicine, Durham, North Carolina, USA; s Department of Medicine, Massachusetts General Hospital and Harvard Medical School, Boston, Massachusetts, USA

**Keywords:** frailty, HIV, major adverse cardiovascular event, statin

## Abstract

**Background:**

People with HIV (PWH) are at increased risk for atherosclerotic cardiovascular diseases (ASCVDs) and geriatric syndromes, including frailty. REPRIEVE (Randomized Trial to Prevent Vascular Events in HIV) demonstrated a 36% reduction in major adverse cardiovascular events (MACE) with pitavastatin vs placebo but the role of statins for ASCVD prevention among frail PWH is not known.

**Objectives:**

The purpose of this study was to evaluate whether frailty is associated with MACE and whether pitavastatin prevents MACE regardless of frailty.

**Methods:**

We conducted a post hoc analysis of the REPRIEVE trial, a randomized, blinded trial of pitavastatin 4 mg vs placebo. Participants included PWH without ASCVD, aged 40 to 75 years. Frailty was measured with a 32-item frailty index. Cox proportional hazards models were used to estimate cause-specific hazard of MACE by frailty status. Pitavastatin effect modification by frailty status was assessed via interaction with treatment.

**Results:**

Of 7769 REPRIEVE participants, 7,740 (>99%) had sufficient data to calculate frailty index. Median age was 50 years (Q1, Q3: 45, 55), 67% were nonfrail, 29% prefrail, and 4% frail at baseline; median follow-up was 5.6 years. Adjusted for age, sex, ASCVD risk score, and treatment group, MACE hazard increased with frailty (*P* < 0.0001): HR: 1.76 (95% CI: 1.35-2.30) among prefrail, and 2.14 (95% CI: 1.33-3.45) among frail compared to nonfrail. There was no evidence that pitavastatin effect differed by frailty status (*P* = 0.44).

**Conclusions:**

Frailty was associated with markedly higher hazard of MACE. Though frailty did not appear to modify the protective effects of pitavastatin seen in the primary trial, the efficacy in frail PWH remains uncertain due to the limited number of frail individuals.

People with HIV (PWH) are at increased risk for atherosclerotic cardiovascular diseases (ASCVDs) compared to people without HIV, even after accounting for traditional risk factors. There is also evidence of accelerated aging among PWH: frailty, a state of physiologic vulnerability, occurs at a younger age and is more prevalent compared to persons without HIV.^1^ Older adults who are frail are at the highest risk of experiencing cardiovascular events,^2^^,^^3^ yet adults with frailty are routinely underprescribed key preventive therapies such as statins due to a lack of data to guide treatment and perceived risk from statins.^4^, ^5^, ^6^

Current risk scores used to estimate risk for future ASCVD events and to guide decisions for preventive therapy, such as statins, may underestimate risk for subgroups of PWH. In the REPRIEVE (Randomized Trial to Prevent Vascular Events in HIV), the American College of Cardiology/American Heart Association Pooled Cohort Equations underestimated ASCVD risk among participants from high-income regions, particularly women and Black participants.^7^ Furthermore, although the REPRIEVE trial demonstrated a 36% reduction in major adverse cardiovascular events (MACE) with pitavastatin, an incidence rate of 4.95 events per 1,000 person-years remained in the pitavastatin group.^8^^,^^9^ Together, the underprediction of ASCVD risk calculators among subgroups of PWH and continued events among participants on pitavastatin suggest the risk is not fully explained by existing tools.

Given the increased risk of frailty among PWH, REPRIEVE represents a unique opportunity to test the hypotheses that 1) frailty is associated with MACE among PWH, even after accounting for ASCVD risk and 2) pitavastatin prevents MACE across the spectrum of frailty. To evaluate these hypotheses, we constructed a frailty index and verified its predictive validity via assessment of its relationship with mortality.

## Methods

REPRIEVE (NCT02344290) was a prospective, double-blind, placebo-controlled trial to evaluate pitavastatin calcium 4 mg daily for prevention of MACE among PWH on antiretroviral therapy.^8^, ^9^, ^10^ Briefly, 7,769 participants were enrolled from March 2015 to July 2019 at over 100 sites in 12 countries, including the United States. Key inclusion criteria were PWH ≥40 and ≤ 75 years of age, on stable combination antiretroviral therapy for at least 6 months, with a CD4 + T-cell count >100 cells/mm,^3^ and low-to-moderate risk for ASCVD based on estimated risk ≤15% using 2013 American College of Cardiology/American Heart Association Pooled Cohort Equations in conjunction with low-density lipoprotein cholesterol meeting specific thresholds depending on the ASCVD risk score. Key exclusion criteria included known ASCVD, diabetes with low-density lipoprotein cholesterol ≥70 mg/dL, impaired renal function, decompensated cirrhosis, active cancer, and ongoing statin use. REPRIEVE participants were followed until the trial was stopped for demonstrated efficacy in March 2023, with closeout visits performed by August 2023.^8^^,^^9^

### Ethics

Each clinical research site obtained Institutional Review Board/ethics committee approval and any other applicable regulatory entity approvals. Written, informed consent was obtained on all participants.

### Primary exposure variable: frailty

The main exposure variable for hypothesis 1 was frailty, characterized according to the accumulation of deficits approach; frailty was evaluated as an effect modifier for hypothesis 2. We leveraged a 32-item frailty index, constructed from data captured in REPRIEVE for assessment of frailty incidence, to evaluate associations between pre-existing frailty at baseline as a prognostic factor of MACE. Following a standard procedure,^11^ items were derived from questions on the Duke Activity Status Instrument,^12^ Rapid Eating Assessment for Participants,^13^ a REPRIEVE muscle symptom questionnaire, vital signs, comorbidities, medications, and laboratory values reported at enrollment. Each binary item of the Frailty Index was coded as “0” if deficit was absent and “1” if deficit was present. For items based on continuous and ordinal variables, an intermediate value was coded as 0.5 (Supplemental Table 1). The REPRIEVE frailty index was calculated as the sum of the scores across items, divided by the total number of nonmissing items, yielding a score from 0 (absence of all deficits) to 1 (presence of all deficits). Frailty status was defined as frailty <0.1 (nonfrail), 0.1 to 0.2 (prefrail), and >0.2 (frail).^14^, ^15^, ^16^ Participants with data available on at least 25 items (∼80% of all items) were included.

In an exploratory analysis, we also investigated the physical phenotype of frailty in a substudy of REPRIEVE. Participants at U.S. Advancing Clinical Therapeutics Globally for Infectious Diseases sites had an opportunity to coenroll in the PREPARE (Pitavastatin to REduce Physical Function Impairment and Frailty in HIV) ancillary study within 24 months of REPRIEVE entry with target sample size of 600.^17^ For PREPARE participants, in addition to frailty status based on the cumulative deficits approach, the physical phenotype of frailty as described by Fried^18^^,^^19^ was captured (subsequently referred to as Frailty Phenotype) based on the following components: 1) weight loss (self-report of unintentional weight loss of 10 or more pounds in the prior year); 2) exhaustion (experiencing at least 3 to 4 times per week the feeling that “everything I do is an effort” or “sometimes I cannot get going”; 3) low physical activity (being “limited a lot” in response to the Short Form 36 question “does your health limit you in vigorous activities such as running, lifting heavy objects, or participating in strenuous sports?”; 4) slow gait by average of two 4-m walk times; and 5) weak grip by average of 3 measurements on a handheld Jamar dynamometer (with cut points as previously defined^18^^,^^19^). Participants were classified as nonfrail if they had no components present, prefrail with one or two components present, and frail with three or more components present.

### Primary outcome variable

We used the REPRIEVE primary outcome of MACE, a composite of cardiovascular death, myocardial infarction, hospitalization for unstable angina, stroke, transient ischemic attack, peripheral arterial ischemia, revascularization of a coronary, carotid, or peripheral artery, or death from an undetermined cause. MACE was independently adjudicated by practitioners who were unaware of treatment group assignment.^8^ Death from any cause was a secondary outcome and used to validate the frailty index in the current analysis.

### Statistical analysis

Distributions of frailty measures were characterized using descriptive statistics. Agreement between Frailty Status by frailty index (the frailty measure available for the overall REPRIEVE cohort) and Frailty Phenotype (exploratory, assessed for the subset of participants coenrolled in the PREPARE substudy) was quantified by Kappa statistics and visualized by Kappa plot, in the subset of participants who had both frailty measures available.

As per standard procedure for creating a frailty index,^11^ we verified predictive validity of the constructed frailty index by evaluating the relationship between Frailty Status (covariate) and all-cause death (outcome)^11^ using Cox proportional hazards regression modeling of time-to-death, adjusted for treatment group, and further adjusted for age and sex at birth.

Event incidence was estimated as the number of events divided by total person-years of follow-up, with follow-up time calculated from randomization to the first event of interest or last contact. For hypothesis 1, Frailty Status as a prognostic factor of MACE was evaluated using Cox proportional hazards regression models with time-to-first MACE as the outcome and non-CVD deaths considered competing events and censored, as in the REPRIEVE primary analysis,^8^^,^^10^ adjusted for treatment group (model 1), and further adjusted for age, sex at birth, and ASCVD risk score at enrollment (model 2). Modification of pitavastatin effect by Frailty Status (hypothesis 2) was assessed via interaction with treatment group. The Aalen estimator was used to visualize cumulative incidence over time.

Proportional hazards assumptions were evaluated via interaction with time and visual examination of the failure probability over time as well as standardized score process. Statistical comparisons were performed using 2-sided Wald significance tests with a 5% type I error, and 2-sided 95% CIs are provided. There was no adjustment for multiplicity. The analysis was conducted using SAS software (Version 9.4 for Linux. Copyright © 2016 SAS Institute Inc).

## Results

### Participant characteristics

Of 7769 REPRIEVE participants, 7,740 (>99%) had data available for at least 25 frailty index components and were included in the analysis (Supplemental Figure 1). There were 29 participants excluded due to insufficient data to measure frailty (missing >20% items). In total, 79% of participants had complete data for all 32 components, 20% on 31 components, and 1% on 25 to 30 components. Consistent with the overall trial population, the median age was 50 years (Q1, Q3: 45, 55), ASCVD risk score 4.5% (2.1%, 7.0%), a majority were non-White (65%), and 31% were female. Age and ASCVD risk score increased from nonfrail to frail, as expected, and the proportion of females was higher among the frail participants (driven primarily by higher body mass index and hypertension among females). Other characteristics are shown in Table 1.

**Table 1:** Characteristics of the Participants at Baseline by Frailty Index Status

	Total (N = 7,740)	Nonfrail (FI <0.1) (n = 5,196)	Prefrail (FI 0.1-0.2) (n = 2,228)	Frail (FI >0.2) (n = 316)
Treatment group				
Pitavastatin	3,876 (50%)	2,608 (50%)	1,115 (50%)	153 (48%)
Placebo	3,864 (50%)	2,588 (50%)	1,113 (50%)	163 (52%)
Age (y)				
Median (Q1, Q3)	50 (45, 55)	49 (45, 54)	51 (46, 55)	52 (48, 56)
40-49	3,714 (48%)	2,640 (51%)	960 (43%)	114 (36%)
50-59	3,349 (43%)	2,132 (41%)	1,054 (47%)	163 (52%)
60+	677 (9%)	424 (8%)	214 (10%)	39 (12%)
Sex at birth				
Male	5,325 (69%)	3,640 (70%)	1,510 (68%)	175 (55%)
Female	2,415 (31%)	1,556 (30%)	718 (32%)	141 (45%)
Gender identity				
Cisgender	7,362 (95%)	4,961 (95%)	2,109 (95%)	292 (92%)
Transgender spectrum	127 (2%)	76 (1%)	43 (2%)	8 (3%)
Not reported	251 (3%)	159 (3%)	76 (3%)	16 (5%)
Race^a^				
Black or African American	3,196 (41%)	2070 (40%)	970 (44%)	156 (49%)
White	2,692 (35%)	1827 (35%)	766 (34%)	99 (31%)
Asian	1,138 (15%)	791 (15%)	312 (14%)	35 (11%)
Other	714 (9%)	508 (10%)	180 (8%)	26 (8%)
Hypertension^b^	2,770 (36%)	956 (18%)	1,549 (70%)	265 (84%)
Diabetes mellitus	37 (<0.5%)	4 (<0.5%)	20 (1%)	13 (4%)
ASCVD risk score (%)				
Median (Q1, Q3)	4.5 (2.1, 7.0)	3.9 (1.7, 6.4)	5.4 (3.0, 8.1)	6.5 (4.3, 8.9)
Body mass index (kg/m^2^)				
Median (Q1, Q3)	25.8 (22.7, 29.4)	24.9 (22.2, 27.9)	27.7 (24.3, 31.5)	31.0 (26.1, 36.0)
CD4 count (cells/mm^3^)				
<500	2,497 (32%)	1,536 (30%)	837 (38%)	124 (39%)
500+	5,243 (68%)	3,660 (70%)	1,391 (62%)	192 (61%)
<LLQ	5,242 (88%)	3,520 (88%)	1,510 (86%)	212 (84%)
HIV-1 RNA (copies/mL)^c^				
LLQ -< 400	616 (10%)	384 (10%)	197 (11%)	35 (14%)
400+	129 (2%)	83 (2%)	40 (2%)	6 (2%)

ASCVD = atherosclerotic cardiovascular disease; FI = frailty index.

Frequency (%) for categorical measures, median with lower and upper quartiles (Q1, Q3) for continuous measures. All statistics are calculated out of participants with data collected. Missing data include: BMI (n = 7), HIV-1 RNA (n = 1,753).

a “Other” race includes participants self-identifying as native or indigenous to the enrollment region; more than one race (with no single race noted as predominant); or of unknown race.

b Hypertension is defined as any of the following: hypertension diagnosis, use of antihypertensive treatment for elevated blood pressure, systolic blood pressure ≥140 mm Hg, diastolic blood pressure ≥90 mm Hg.

c HIV-1 RNA was captured if available through standard of care. The assays used for testing varied, including assays with lower limits of quantitation (LLQ) between 20 and 400 copies/mL.

### Characterization of frailty

**Figure 1:**
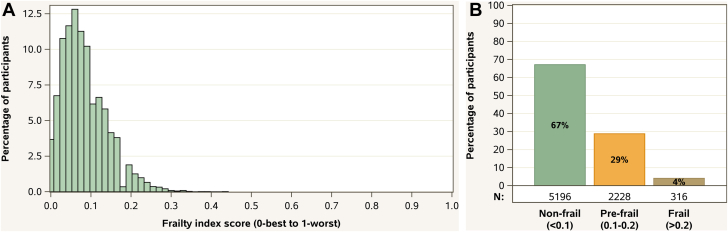
**Frailty Index Distribution** (A) Distribution of frailty index scores, where <0.1 indicates nonfrail, 0.1 to 0.2 prefrail, and >0.20 frailty. (B) Frailty categorization, by proportion of nonfrail, prefrail, and frail participants in REPRIEVE. REPRIEVE = Randomized Trial to Prevent Vascular Events in HIV.

The distribution of frailty index scores is shown in Figure 1A and contributions of individual items in Supplemental Figure 2; 67% of participants were classified as nonfrail (n = 5,196), 29% as prefrail (n = 2,228), and 4% as frail (n = 316) (Figure 1B). The maximum observed frailty score was 0.44, with most participants classified as frail having a score between 0.20 and 0.25.

### Relationship between frailty status and mortality

To evaluate the predictive validity of the constructed frailty index, we examined frailty status as a prognostic factor of all-cause mortality based on a median of 5.6 years of follow-up and 269 deaths observed in REPRIEVE. There was a strong association between frailty and hazard of death (*P* < 0.0001) (Supplemental Figure 3). The hazard of death was higher among prefrail (HR: 1.72; 95% CI: 1.34-2.20) compared to nonfrail participants. A similar trend was observed comparing frail to nonfrail, with a wide CI due to small numbers of participants and deaths among the frail (13 deaths among n = 316; HR: 1.51; 95% CI: 0.85-2.65). The results were robust to adjustment for age and sex at birth, confirming the association of the constructed index with mortality beyond age, and accounting for aging process varying by sex.

### Frailty status as a prognostic factor of mace

**Figure 2:**
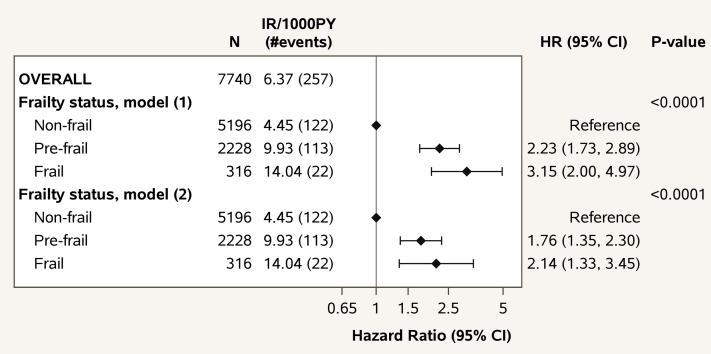
**Frailty Status as a Prognostic Factor of MACE** Cause-specific HR estimates are from Cox proportional hazards models with baseline frailty status as a covariate, adjusted for treatment group (1), and for treatment group, age, sex at birth, and ASCVD score at enrollment (2). Non-CV deaths without MACE were treated as competing events. Type 3 *P* values for the overall effect of frailty status are shown. For visual purposes, HR with CI are shown in the log scale. ASCVD = atherosclerotic cardiovascular disease; IR = incidence rate of MACE; MACE = major adverse cardiovascular event; N = number of participants at risk; PY = person-years of follow-up, #events = number of MACE endpoints.

Next, we evaluated frailty status as a prognostic factor of the REPRIEVE primary MACE endpoint (Figure 2, Supplemental Figure 4). Overall, 257 MACE endpoints were observed: 122 among the nonfrail, 113 among the prefrail, and 22 among the frail. MACE hazard was substantially higher with frailty (*P* < 0.0001), robust to adjustment for age, sex at birth, and enrollment ASCVD risk score. There was a marked 76% increase in MACE endpoints among the prefrail compared to the nonfrail (HR: 1.76; 95% CI: 1.35-2.30), and 114% increase among the frail compared to the nonfrail (HR: 2.14; 95% CI: 1.33-3.45) participants.

### Exploratory analyses of frailty phenotype

Among the subset of participants who also completed frailty assessments using the Frailty Phenotype (n = 589), 54% were nonfrail (n = 315), 41% as prefrail (n = 242), and 5% as frail (n = 32). We assessed agreement with status by the frailty index (Supplemental Figure 5). Despite differences in frailty constructs, 57% of observations were in exact agreement, 40% in partial agreement, and 3% not in agreement (weighted Kappa 0.24), consistent with the level of agreement reported by others.^20^ With respect to relationship to MACE, the association between Frailty Phenotype and MACE was less pronounced than with the frailty index, albeit based on limited numbers of participants and only 33 MACE endpoints (Supplemental Figure 6).

### Frailty status as a statin effect modifier

**Figure 3:**
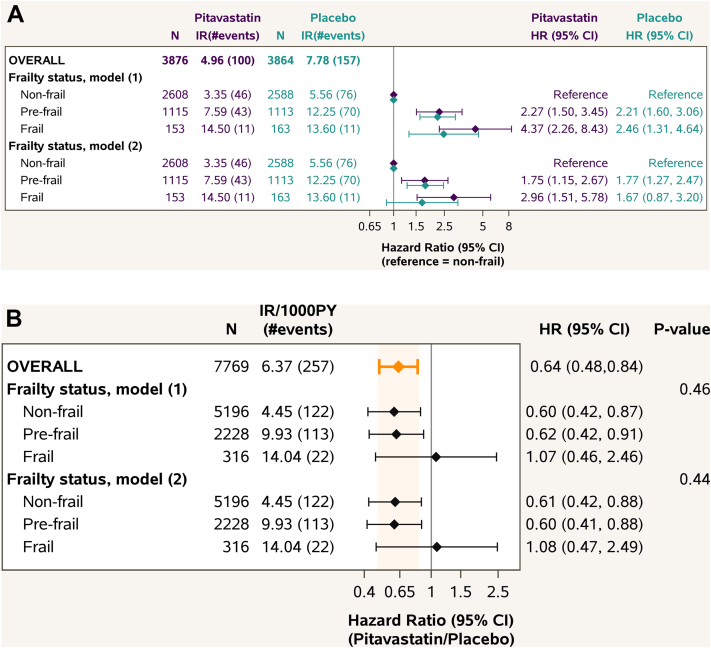
**Pitavastatin Effect in Subgroups by Frailty Status** (A) MACE by Frailty Status Within Each Treatment Group.(B) Pitavastatin Effect on MACE by Subgroups of Frailty Status Estimates are from Cox proportional hazards models with baseline frailty status, treatment group, and their interaction as covariates (model 1), and further adjusted for age, sex at birth, and ASCVD score at enrollment (model 2). Type 3 *P* values for the interaction between frailty and treatment group are shown. For visual purposes, HR with CI are shown in the log scale. For reference, the overall pitavastatin effect observed in REPRIEVE is shown on the top (in orange). Abbreviations as in Figures 1 and 2.

In the REPRIEVE trial, a 36% reduction in the MACE endpoints with pitavastatin was observed.^8^, ^9^, ^10^ As shown in Figure 3, there was no evidence that this pitavastatin effect differed across subgroups by frailty status (*P* = 0.44). The data in the frail group were nonconclusive due to only 316 participants and 22 MACE endpoints (11 in each treatment group) and wide CI (95% CI: 0.47-2.49), and appeared driven by the lower MACE incidence among the frail compared to prefrail participants in the placebo group (Figure 3A).

## Discussion

In a post hoc analysis of the REPRIEVE trial, prefrailty was present in 29%, even in this relatively young and clinically stable cohort of PWH, and 4% were identified as frail. Higher levels of frailty were predictive of mortality beyond age, confirming validity of the constructed frailty index. Frailty was strongly associated with increased risk of MACE, independent of age, sex at birth, and ASCVD risk score. These findings highlight frailty as a potential additional risk factor for MACE among PWH, even in a fairly young population with only low-to-moderate ASCVD risk. Importantly, frailty status did not appear to modify the protective effects of pitavastatin seen in the primary trial, although the results among the frail were inconclusive due to limited numbers of frail PWH and events (Central Illustration).

**Central Illustration:**
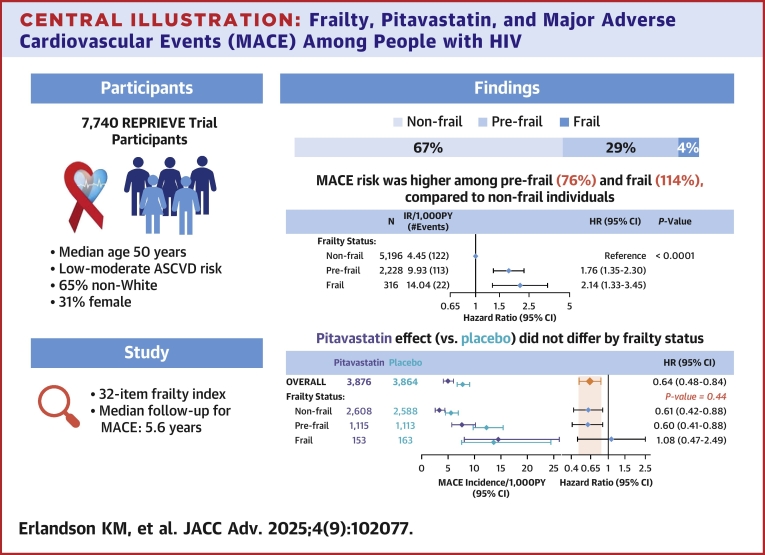
**Frailty, Pitavastatin, and Major Adverse Cardiovascular Events (MACE) Among People with HIV** We developed a 32-item frailty index among 7,740 people with HIV in the REPRIEVE trial. Participants had a median age of 50 years; 29% were considered prefrail and 4% frail. MACE risk was higher among prefrail and frail participants compared to frail individuals. The pitavastatin effect did not differ by frailty status, although conclusions were limited in the frail group due to small number of frail individuals. Abbreviations as in Figures 1 and 2.

REPRIEVE represents an important step forward to understanding the role of pitavastatin as a preventive strategy for ASCVD among PWH who appear to experience premature aging. In REPRIEVE, about a third of the population were identified as prefrail or frail, similar to other cohorts of PWH with rates of frailty from 3% to 70% and prefrailty from 10% to 63%, and substantial variation seen by living environment and baseline health.^21^ Importantly, prefrail and frail individuals had the highest risk of MACE and mortality, consistent with prior evidence.^22^ Although we did not find that relative statin effect differed by frailty status, our assessment may have been underpowered. In a cohort of over 710,000 U.S. Veterans aged 65 and older, frailty was similarly associated with a higher risk of MACE, and while new statin-users who were frail had greater absolute risk reduction for MACE compared to those who were nonfrail, there was no evidence of effect modification by frailty.^23^

No apparent differential pitavastatin benefit by frailty status is a key finding of our study. Clinicians may question the benefit of statin therapy among prefrail or frail PWH, particularly in the context of multimorbidity and polypharmacy. Although our findings were not conclusive among the frailest individuals due to small numbers, our findings support the use of statins for cardiovascular prevention, regardless of frailty status among PWH. A similar phenomenon has been reported in several heart failure trials that have found the same or even greater benefit of therapeutic interventions in older adults with frailty.^24^^,^^25^ We hypothesize that because older adults with prefrailty or frailty are at increased risk of cardiovascular outcomes, they may also therefore be most likely to benefit from a cardiovascular targeted intervention.

Traditional risk scores may not accurately identify PWH at high risk for ASCVD events. As a measure of both chronologic age and a novel modifiable risk factor for ASCVD, frailty (by any validated definition) may further inform ASCVD risk prediction in PWH.^26^^,^^27^ A prior study among 944 PWH found that frailty (by phenotype) was an independent predictor of ASCVD events though did not improve discrimination of the Pooled Cohort Equations, possibly due to a limited number of events (n = 39 compared to 257 in REPRIEVE).^28^^,^^29^ Importantly, many frailty definitions, including ours, include modifiable factors that could influence subsequent ASCVD risk, such as obesity, weakness, low physical activity, polypharmacy, or smoking-related conditions. In a general population, improvement in frailty has been shown to lower subsequent ASCVD risk.^27^

Frailty, measured according to the frailty index, was strongly associated with increased risk of MACE. A similar pattern was observed by Frailty Phenotype, though limited by a smaller sample size. The frailty index, defined by Rockwood and colleagues, posits that deficits across multiple domains of health accumulate over the life span, reflecting increased vulnerability.^11^ The Veterans Aging Cohort Index reflects a similar deficit accumulation and has been used in populations of PWH; however, many variables included in this index were not collected in REPRIEVE.^30^ Distinct from this approach, Fried and colleagues describe a phenotype of frailty related to physical function.^19^ Notably, the Frailty Phenotype is collected prospectively and requires approximately 10 to 15 minutes to complete while the Rockwood frailty index approach can utilize existing data often already collected in the context of clinical trials and could be calculated using machine learning rather than requiring personnel time. Thus, if a frailty measure were to be adopted as part of routine clinical practice, the frailty index may be more feasible.^31^^,^^32^ Each frailty definition is based on a different underlying theory, both are predictive of adverse outcomes and sensitive to change, and each identified a somewhat different group of frail individuals, consistent with other studies that find similar results when both frailty definitions are available.^33^^,^^34^ Taken together, these results should encourage clinicians and researchers to use any validated frailty assessment to assess frailty.

This study has important strengths. REPRIEVE was a multinational, double-blind, randomized controlled trial. About one-third of our study population were female, a population often underrepresented in cardiovascular trials of PWH. We were able to measure frailty using the 2 leading definitions.

### Study Limitations

The study cohort is relatively healthy and may not reflect the general population of PWH. Sample size was limited in the frail group, and more data are needed to confirm statin effect in this group. Socioeconomic status was not collected and thus could not be accounted for in the analysis. Targeted collection of medications may have underestimated the burden of polypharmacy; dementia/cognitive impairment were collected by self-report or known diagnosis and likely also underestimated more mild impairments. REPRIEVE did not enroll adults over age 75, and only 9% of participants were aged 60 or older, so the benefit of pitavastatin in this population remains unknown. Nonetheless, approximately 1/3 were prefrail or frail in a relatively younger population, and frailty strongly predicted MACE among a group with low-to-moderate cardiovascular risk, of relevance to the large global population of asymptomatic PWH at risk for cardiovascular disease.

## Conclusions

In this post hoc analysis of REPRIEVE, higher levels of frailty were associated with increased risk of MACE. Incorporating frailty assessment into the routine care of PWH may help identify those at further risk of ASCVD who may otherwise be missed with routine screening tools. The protective effects of pitavastatin seen in the primary trial were evident among nonfrail and prefrail PWH, though the efficacy in frail PWH remains uncertain due to the limited number of frail individuals in this study.

Perspectives **COMPETENCY IN MEDICAL KNOWLEDGE AND SYSTEMS-BASED PRACTICE:** Incorporating a simple, pragmatic frailty assessment into the routine care of PWH may help identify those at further risk of ASCVD who may otherwise be missed with routine screening tools.

**TRANSLATIONAL OUTLOOK:** Importantly, many frailty definitions, including ours, include modifiable factors that could influence subsequent ASCVD risk (obesity, weakness, low physical activity, polypharmacy), and improvement in frailty may reduce subsequent ASCVD risk. Lastly, clinicians may question the benefit of statin therapy among prefrail or frail PWH, particularly in the context of multimorbidity and polypharmacy. Although our findings were not conclusive among the frailest individuals due to small numbers, our findings support the use of statins for cardiovascular prevention, regardless of frailty status among PWH.

## Funding support and author disclosures

This study is supported through 10.13039/100000002NIH grants U01HL123336 and 1UG3HL164285, to the Clinical Coordinating Center, and U01HL123339 and 1U24HL164284, to the Data Coordinating Center, R01AG054366 as well as funding from Kowa Pharmaceuticals America, Inc, 10.13039/100005564Gilead Sciences, and ViiV Healthcare. The 10.13039/100000060NIAID supported this study through grants UM1 AI068636, which supports the ACTG Leadership and Operations Center; and UM1 AI106701, which supports the ACTG Laboratory Center. This work was also supported by the 10.13039/100016758Nutrition Obesity Research Center at Harvard (P30DK040561 to SKG). The 10.13039/100000002National Institutes of Health supported the study and had a role in study design and oversight of the study. Kowa Pharmaceuticals America, Inc, Gilead Sciences, and ViiV Healthcare provided financial support for the study but did not have a role in study design, collection of data, analysis, writing, or decision to publish. Dr Erlandson has received grants from 10.13039/100000049NIH/NIA during the conduct of the study, as well as a grant from Gilead and consulting payments from Gilead, ViiV, and Merck (all to her employer) outside of the submitted work. Dr Umbleja has received grants from 10.13039/100000050NIH/NHLBI, Kowa Pharmaceuticals, and 10.13039/100000049NIH/NIA during the conduct of the study, as well as grants from 10.13039/100000060NIH/NIAID, outside the submitted work. Dr Ribaudo has received grants from Kowa Pharmaceuticals during the conduct of the study, as well as grants from 10.13039/100000060NIH/NIAID, 10.13039/100000050NIH/NHLBI, 10.13039/100000062NIH/NIDDK, and 10.13039/100000049NIH/NIA, outside of the submitted work. Dr Zanni has received grant support through her institution from 10.13039/100000060NIH/NIAID and Gilead Sciences, Inc, relevant to the conduct of the study, as well as grants from 10.13039/100000060NIH/NIAID and 10.13039/100000050NIH/NHLBI; support for attending CROI and International Workshop for HIV and Women from conference organizing committee when abstract reviewer and/or speaker; and participation in DSMB for NIH funded studies, outside the submitted work. Dr Fichtenbaum has received research grant support through his institution from Gilead Sciences, ViiV Healthcare, GSK, and Merck, all outside the submitted work. Dr Malvestutto has received institutional research support by Lilly and honoraria from ViiV Healthcare, Gilead Sciences, and Pfizer for advisory board membership, all outside the submitted work. Dr Aberg has received institutional research support for clinical trials from Emergent Biosolutions, Gilead Sciences, Glaxo Smith Kline, Janssen, Merck, Pfizer, Regeneron, and ViiV Healthcare and personal fees for advisory boards from Glaxo Smith Kline/ViiV and Merck; and participation on DSMB for Kintor Pharmaceuticals, all outside the submitted work. Dr Currier has received consulting fees from Merck and Company and Resvirlogix, outside the submitted work. Dr Heath has received institutional research support for clinical trials from Abbvie, Gilead Sciences, Glaxo Smith Kline, Merck, Regeneron, and Viiv Healthcare; and also participates on DSMB for Pfizer. All activities are outside the scope of the submitted work. Dr Lu has received grant support through his institution from the 10.13039/100000050NIH/NHLBI and Kowa Pharmaceuticals America for the conduct of the REPRIEVE trial; and also has received research support to his institution from the American Heart Association, AstraZeneca, Ionis, Johnson & Johnson Innovation, MedImmune, the National Academy of Medicine, the 10.13039/100000050NIH/NHLBI, and the Risk Management Foundation of the Harvard Medical Institutions Incorporated outside of the submitted work. Dr Grinspoon has received grant support through his institution from NIH, Kowa Pharmaceuticals America, Inc, Gilead Sciences, Inc, and ViiV Healthcare for the conduct of the study; personal fees from Theratechnologies and ViiV; and has served on the Scientific Advisory Board of Marathon Asset Management, all outside the submitted work. The views expressed in this manuscript are those of the authors and do not necessarily represent the views of the National Heart, Lung, and Blood Institute or the National Institute of Allergy and Infectious Diseases; the National Institutes of Health; or the U.S. Department of Health and Human Services. All other authors have reported that they have no relationships relevant to the contents of this paper to disclose.
