# Supplementary material for: Frailty, Pitavastatin, and Major Adverse Cardiovascular Events Among People With HIV
Source: JACC Adv. 2025 Aug 20;4(9):102077. doi: 10.1016/j.jacadv.2025.102077 (PMC12539451; doi:10.1016/j.jacadv.2025.102077)
Supplement: Supplementary Material [file mmc1.pdf]

Supplementary Appendix to Erlandson KM, et al.

The Role of Frailty and Pitavastatin for Atherosclerotic Cardiovascular Disease Prevention Among People With HIV.

This appendix has been provided by the authors to give readers additional information about the work.

Table of Contents

1 DATA COLLECTION AND DEFINITIONS **3**

2 FRAILTY INDEX ITEMS AND CODING **5**

Table of Tables

Table S1: Definition of Frailty Index Items 5

Table of Figures

Figure S1: Participant Flow Diagram 9
Figure S2: Contributions of Individual Frailty Index Items 10
Figure S3: Agreement between Frailty Status and Frailty Phenotype 11
Figure S4: Association between Frailty Status and Mortality 11
Figure S5: Cumulative Incidence of MACE Over Time by Frailty Status 12
Figure S6: Association between Frailty Phenotype and MACE in PREPARE 12

1 Data Collection and Definitions

Data collection related to Frailty Index components is described below.

DASI

Duke Activity Status Index (DASI) questionnaire described by Hlatky et al. *[Am J Cardiol. 1989 Sep 15;64(10):651-4.]* self-administered at REPRIEVE entry.

REAP

Rapid Eating and Activity Assessment for Patients (REAP) questionnaire described by Gans et al. *[J Nutr Educ Behav.* ***2006****; 38(5):286–292]* self-administered at REPRIEVE entry.

Myalgia Symptom Assessment

Muscle ache and weakness assessments were conducted as participant interviews by the site staff at study entry. Participants were asked to assess any symptoms as follows.

**Grading of Muscle Ache and Weakness**	
**Grade 0:**	Not experiencing muscle aches (or weakness)
**Grade 1:**	Causing no or minimal interference with usual social and functional activities
**Grade 2:**	Causing greater than minimal interference with usual social and functional activities
**Grade 3:**	Causing inability to perform usual social and functional activities
**Grade 4:**	Disabling: causing inability to perform basic self-care functions or impairing breathing

Anthropometric Measurements and Vital Signs:

Height, weight and blood pressure were measured as part of physical exam at screening (within 90 days prior to study entry). Body mass index (BMI) was calculated based on weight and height using a standard formula, and categorized according to the WHO race-specific cutoffs (18.5, 23 and 27.5 kg/m^2 for Asian race, and 18.5, 25, 30 kg/m^2 otherwise) *[WHO Expert Consultation. Appropriate body-mass index for Asian populations and its implications for policy and intervention strategies. Lancet. 2004 Jan 10;363(9403):157-63. doi: 10.1016/S0140-6736(03)15268-3. Erratum in: Lancet. 2004 Mar 13;363(9412):902. PMID: 14726171].*

Pre-Existing Medical Conditions

Participants were asked regarding the presence of the select following pre-existing medical conditions: hypertension, diabetes mellitus, AIDS-defining events *[CDC. Revised Surveillance Case Definition for HIV Infection -- United States, 2014. MMWR Recomm Rep. 2014;63:1–10],* any malignancy (exclusive of basal/squamous cell skin cancer), dialysis or renal transplantation, chronic active hepatitis B, chronic active hepatitis C, venous thromboembolism, and pulmonary thromboembolism. History of depression was summarized as a participant's self-report of ever having been treated for depression with medication.

Fasting Glucose

Fasting plasma glucose from sodium fluoride/potassium oxalate tubes were collected at entry and stored at ultrafreezer temperatures in a central repository for batch testing by Quest Diagnostics (Baltimore, MD and Teterboro/Clifton, NJ) using commercially available US Food and Drug Administration cleared methods. Glucose level was quantified using a hexokinase technique. Fasting was defined as nothing to eat or drink except water and required prescription medications for at least 8 hours.

Laboratory Values

Hemoglobin, platelets, creatinine and CD4+/CD8+ T cell count were performed as part of the study or obtained from clinical care (if available within 180 days prior to entry for CD4+/CD8+ T cell count; if available within 90 days prior to entry otherwise). Kidney function was estimated via glomerular filtration rate (eGFR) calculated using Chronic Kidney Disease Epidemiology Collaboration (CKD-EPI).

Medications

Participants' current medications were recorded for targeted categories including: antiretroviral, antihypertensive, antidiabetic, anticoagulant and aspirin, antihepatitis, non-statin lipid-lowering drugs and hormonal. Antihypertensive medications prescribed for off-label use were not recorded.

2 Frailty Index Items and Coding

All data were captured prior to study treatment initiation, either at REPRIEVE screening or entry. See Section 1 above for details on data collection.

Table S1: Definition of Frailty Index Items

ID	Source	Variable		Coding
FUNCTION				
1	DASI, Question 1	Able to take care of self (ADLs)	Coded based on participant's response to the given questionnaire question at entry.	Yes = 0, No = 1
2	DASI, Question 6)	Do light work around the house (IADL)		
3	DASI, Question 7	Do moderate work around the house (IADL)		
4	DASI, Question 2	Walk indoors		
5	DASI, Question 3	Walk 1-2 blocks		
6	DASI, Question 4	Climb a flight of stairs		
7	DASI, Question 11	Participate in moderate recreational activities		
8	REAP, Question Q29	Trouble being able to shop or cook? (IADL)	Coded based on participant's response to the given questionnaire question at entry.	Yes = 1, No = 0
9	Muscle symptom questionnaire	Muscle weakness	Coded based on participant-reported grade at entry.	Grade 0 = 0<br>Grade 1,2 = 0.5<br>Grade 3,4 = 1
10	Muscle symptom questionnaire	Muscle aches		
VITALS				
11	Detailed VS	SBP	Coded based on systolic blood pressure measurement from screening.	<140 mmHg = 0<br>≥140 mmHg = 1
12	Detailed VS	DBP	Coded based on diastolic blood pressure measurement from screening.	<90 mmHg = 0<br>≥90 mmHg = 1
13	Detailed VS	BMI	Coded based on BMI calculated using height and weight measurements from screening, and race-specific cut-offs.	For Asian race:<br>BMI ≥18.5 to <23 kg/m^2 = 0<br>BMI ≥23 to <27.5 kg/m^2 = 0.5<br>BMI ≥27.5 kg/m^2 = 1<br>BMI <18.5 kg/m^2 = 1<br>For other races:<br>BMI ≥18.5 to <25 kg/m^2 = 0<br>BMI ≥25 to <30 kg/m^2 = 0.5<br>BMI ≥30 kg/m^2 = 1<br>BMI <18.5 kg/m^2 = 1

MORBIDITIES				
14	Comorbidities/ clinical events	Hypertension	Coded based on presence of hypertension in the medical history. Medical history represents history of hypertension including diagnoses ongoing at entry, and previous diagnoses that have since resolved. All events recorded by the sites are included, including essential hypertension as well as secondary hypertension due to another disorder, drug-induced hypertension, pregnancy-induced hypertension and increased blood pressure as they all may indicate pre-existing issues with blood pressure.	Yes = 1, No = 0
15	Comorbidities/ clinical events	Diabetes	Coded based on presence of diabetes in the medical history. Medical history represents history of diabetes, including current diabetes at entry and previous diagnoses that have since resolved. Note that current diabetes mellitus with LDL-C ≥70 mg/dL was exclusionary per eligibility criteria. Because the protocol required reporting of diabetes mellitus only, cases of steroid-induced or medication-induced diabetes, gestational diabetes, and single elevated blood glucose values were not included.	Yes = 1, No = 0
16	Comorbidities/ clinical events	Heart failure	Based on the REPRIEVE exclusion criteria, assigned as No for all participants at entry.	Yes = 1, No = 0
17	Comorbidities/ clinical events	Atrial fibrillation	Based on central reading of ECG results at entry; coded as Yes if atrial fibrillation was identified from the ECG at entry.	Yes = 1, No = 0
18	Comorbidities/ clinical events	Carotid disease[1]	Exclusionary per REPRIEVE eligibility criteria. Assigned as No for all at entry.	Yes = 1, No = 0
19	Comorbidities/ clinical events	Peripheral vascular disease[1]	Exclusionary per REPRIEVE eligibility criteria. Assigned as No for all at entry.	Yes = 1, No = 0
20	Comorbidities/ clinical events	Cancer (non-AIDS)	Coded based on presence of non-AIDS defining cancer in the medical history. Medical history represents history of cancer. Active cancers within 12 months prior to study entry were exclusionary per eligibility criteria, except successfully treated non-melanomatous skin cancer	Yes = 1, No = 0

[1] *The Frailty Index leveraged in this analysis was constructed for evaluation of frailty in REPRIEVE over time. Two of the items include events also included in the REPRIEVE MACE endpoint definition used as outcome in this analysis. However, as pre-existing CVD was exclusionary at REPRIEVE enrollment, these items did not contribute to the baseline frailty index score which was used in this analysis.*

			and Kaposi's sarcoma without visceral involvement.	
21	Comorbidities/ clinical events	CKD/ESRD	ESRD was defined as dialysis or renal transplantation.<br>Coded based on presence of ESRD in medical history and eGRF from entry (eGFR<60 mL/min/1.73m^2 was considered CKD).	CKD (no ESRD) = 0.5, ESRD = 1;<br>0 otherwise
22	Comorbidities/ clinical events	Liver disease/cirrhosis	Coded based on presence of chronic active HBV, HCV or liver cirrhosis in medical history.<br>Note that known decompensated cirrhosis was exclusionary per eligibility criteria, and those with known active chronic HBV or HCV must have had FIB-4 score ≤3.25 to enroll (>3.25 is an established threshold suggestive of cirrhosis).	Yes = 1, No = 0
23	Comorbidities/ clinical events	Lung disease	While lung disease was not captured as part of medical history, assumed No for all at entry given the REPRIEVE eligibility criteria generally excluding serious illnesses, known active infections and other medical conditions.	Yes = 1, No = 0
24	Comorbidities/ clinical events	Thyroid	While thyroid disease was not captured as part of medical history, assumed No for all at entry given the REPRIEVE eligibility criteria generally excluding serious illnesses, known active infections and other medical conditions.	Yes = 1, No = 0
25	Comorbidities/ clinical events	AIDS defining event	Coded based on presence of AIDS-defining cancer in the medical history.<br>MH represents history of AIDS-defining events, including events reported as ongoing at enrollment.	Yes = 1, No = 0
26	Comorbidities/ clinical events	Anemia	Coded based on the hemoglobin value from screening and the sex-specific thresholds for Grade 3 in the DAIDS Table for Grading the Severity of Adult and Pediatric Adverse Events, corrected Version 2.1, July 2017 used in REPRIEVE.	Males:<br>≥9 g/dL = 0<br><9 g/dL = 1<br><br>Females:<br>≥8.5 g/dL = 0<br><8.5 g/dL = 1
27	Comorbidities/ clinical events	Thrombo-cytopenia	Coded based on the platelet count from screening and the threshold for Grade 3 in the DAIDS Table for Grading the Severity of Adult and Pediatric Adverse Events, corrected Version 2.1, July 2017 used in REPRIEVE.	≥50,000 cells/mm^3 = 0<br><50,000 cells/mm^3 = 1

MOOD/COGNITION				
28	Comorbidities/event	Depression	Coded based on ever being treated for depression with medications per participant self-report.	Yes = 1, No = 0
29	Comorbidities/event	Dementia/ Cognitive impairment	Assumed to be No for all participants at entry, given the exclusion criterion: "Other medical, psychiatric, or psychological condition that, in the opinion of the site investigator, would interfere with completion of study procedures and or adherence to study drug."	Yes = 1, No = 0
LABS				
30	Labs	CD4+/CD8+	Coded based on CD4+/CD8+ ratio calculated based on the values from screening.	≥1 = 0,<br>0.5 - <1 = 0.5<br><0.5 = 1
31	Labs	Fasting glucose	Coded based on the fasting glucose value at entry reported by the central testing lab.	<100 mg/dL = 0<br>100-125 mg/dL = 0.5<br>≥126 mg/dL = 1
MEDICATIONS				
32	Medications	Polypharmacy	Coded based on the agents reportable in REPRIEVE including ARVs; components of FDCs were counted as separate drugs given the potential for interactions. Vaccines were not included.<br>Note that due to the targeted collection of medications, the actual polypharmacy burden is likely much higher.	<5 meds = 0,<br>≥5 meds = 1

Figure S1: Participant Flow Diagram

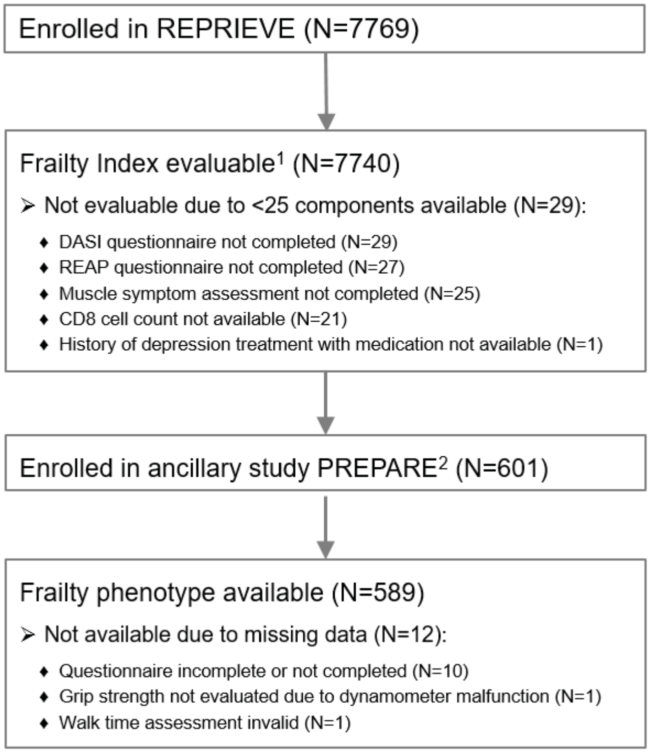

[1] *Participants with data available on at least 25 Frailty Index components were evaluable and included in the analysis.*
[2] *PREPARE was an ancillary study of REPRIEVE to evaluate physical function and frailty, conducted at U.S. ACTG sites.*

Figure S2: Contributions of Individual Frailty Index Items

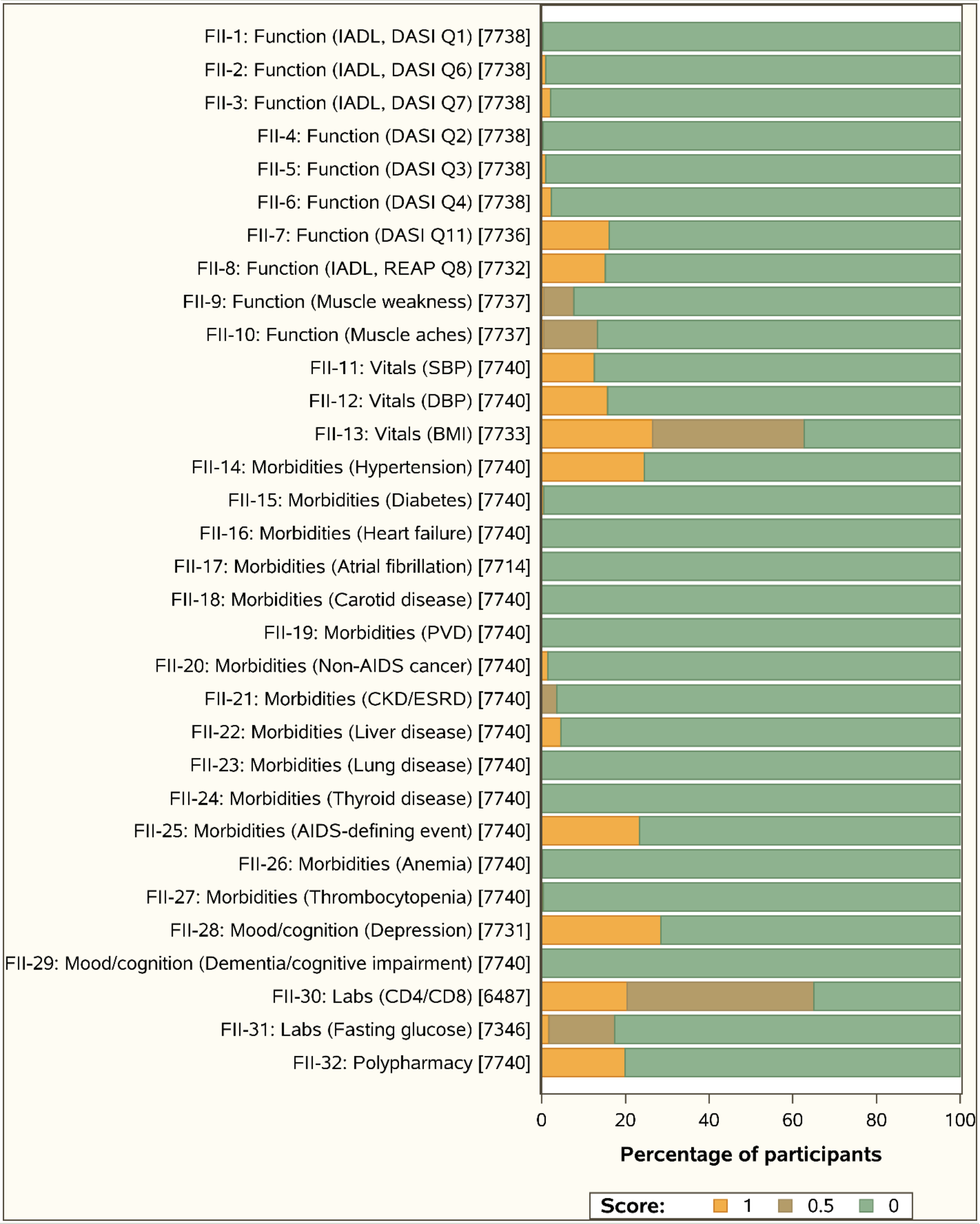

The numbers of participants with data available are shown in brackets after each FI item (FII).
Score of 1 reflects maximum deficits and 0 no deficits observed for a given FI item.

Figure S3: Agreement between Frailty Status and Frailty Phenotype

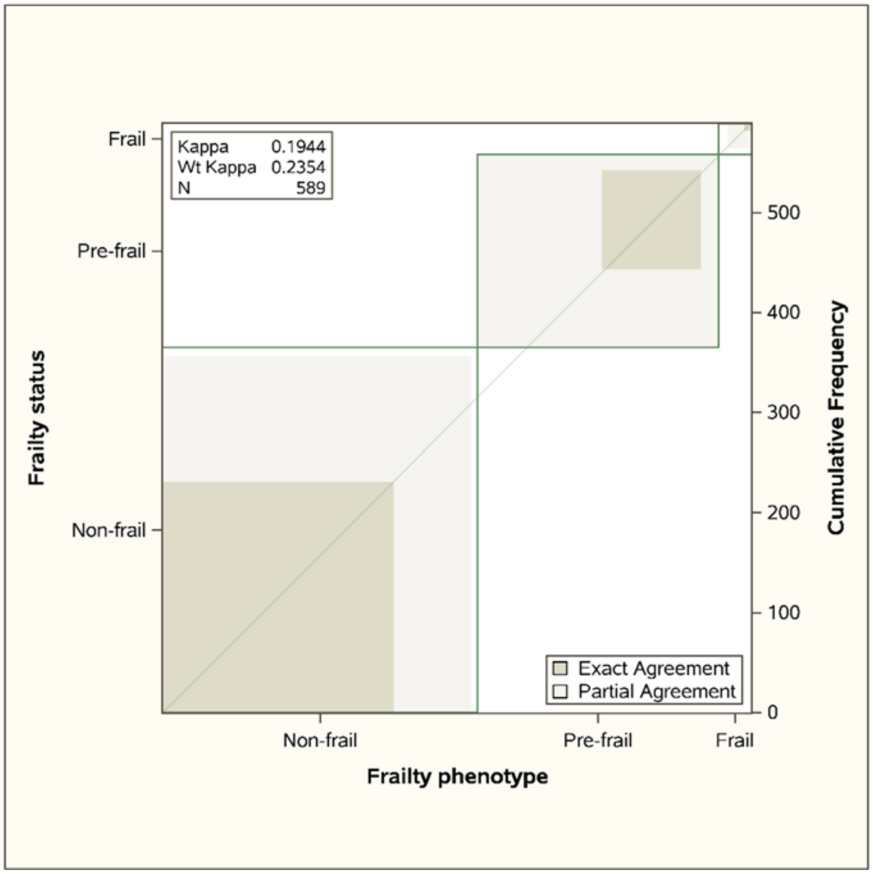

Agreement is visualized using Kappa plot. Exact agreement reflects the exact same category based on Frailty Status and Frailty Phenotype (e.g. frail vs. frail); partial agreement reflects adjacent categories (non-frail vs. pre-frail, pre-frail vs. frail).
Participants who enrolled in the REPRIEVE physical function and frailty ancillary study PREPARE are included. Of those, 45% were enrolled into REPRIEVE and PREPARE concurrently and 55% were enrolled into PREPARE after their REPRIEVE enrollment, mostly within a year. For the latter, the timing of Frailty Index (evaluated at REPRIEVE entry) and Frailty Phenotype (evaluated at PREPARE entry) differs accordingly.
Wt Kappa denotes weighted Kappa statistic, N number of participants.

Figure S4: Association between Frailty Status and Mortality

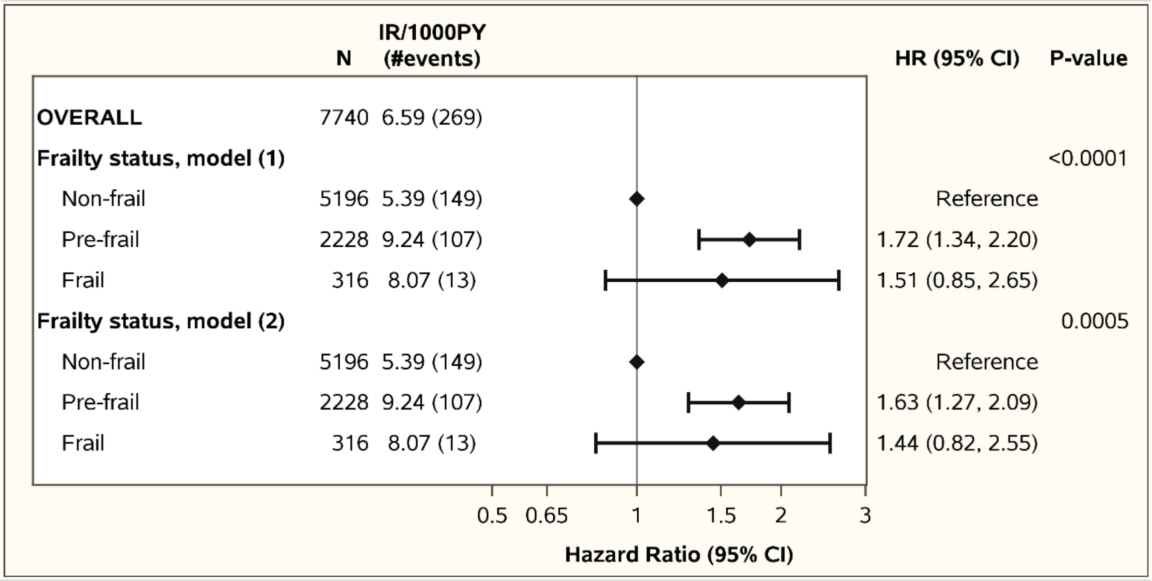

Estimates are from Cox proportional hazards models with baseline frailty status as a covariate, adjusted for treatment group (1), and for treatment group, age and sex at birth (2). Type 3 p-values for the overall effect of frailty status are shown. The Uno's C-statistic was 0.63 for model (1), and 0.68 for model (2), indicating moderate discrimination. For visual purposes, HR with CI are shown in the log scale.

N denotes number of participants at risk, IR incidence rate of death, PY person-years of follow-up, #events number of deaths, HR hazard ratio and CI confidence interval.

Figure S5: Cumulative Incidence of MACE Over Time by Frailty Status

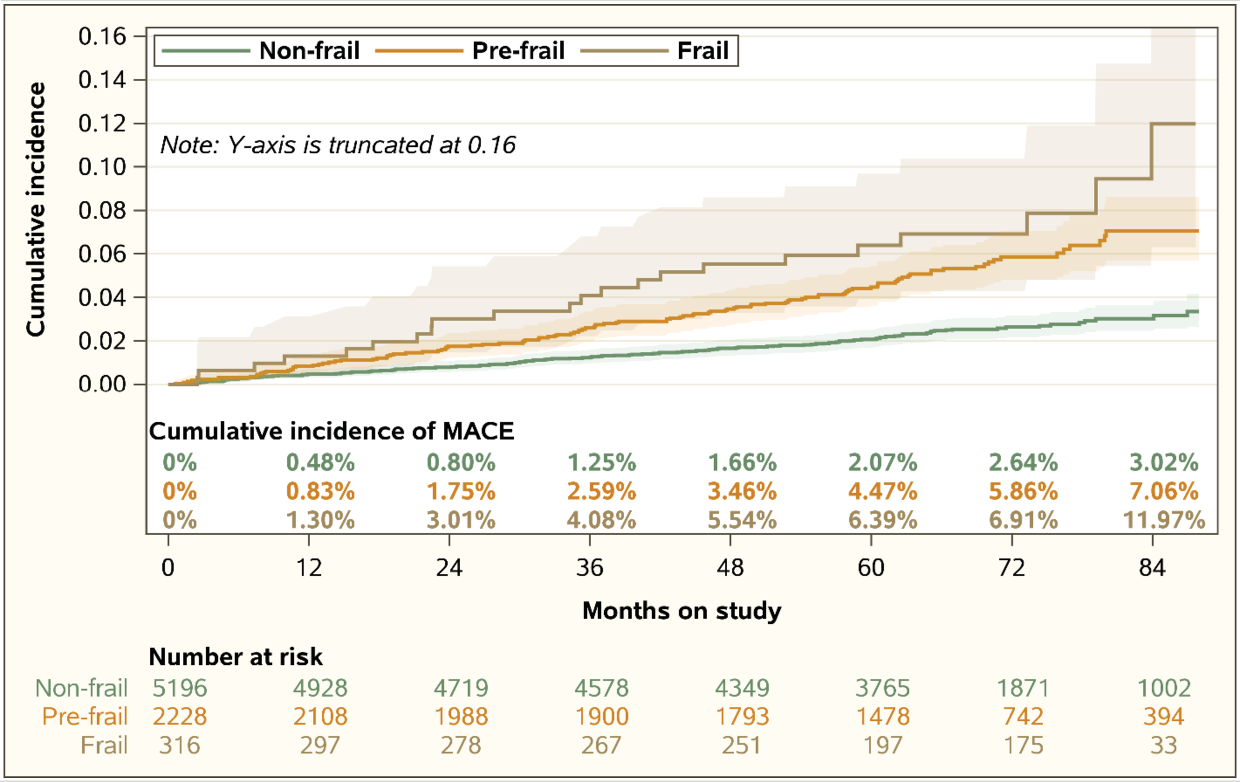

Cumulative incidence was calculated using the Aalen estimator for probability of subdistribution of failure of interest. Participant follow-up was calculated as calendar months (30.44 days) from randomization date to the date of event or last contact, whichever was earlier; participants with no contact after entry were included with 1 day imputed as censoring time.

Figure S6: Association between Frailty Phenotype and MACE in PREPARE

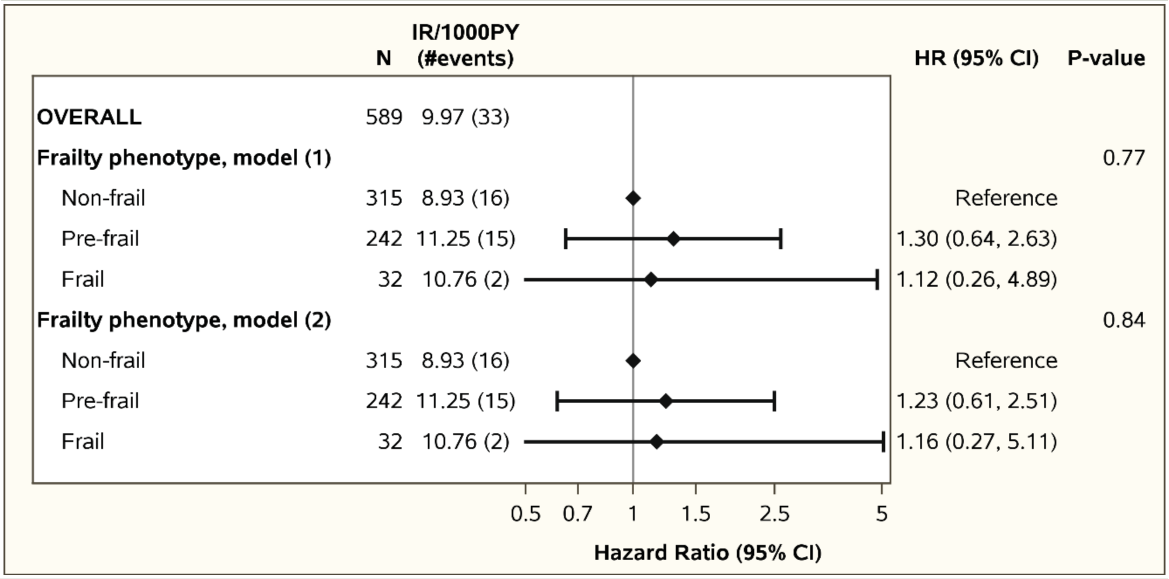

Participants who enrolled in the REPRIEVE physical function and frailty ancillary study PREPARE are included (n=589). Of those, 45% were enrolled into REPRIEVE and PREPARE concurrently and 55% were enrolled into PREPARE after their REPRIEVE enrollment, mostly within a year. For the latter, the observed Frailty Phenotype may not reflect that from prior to study treatment initiation at REPRIEVE entry.

Cause-specific HR estimates are from Cox proportional hazards models with Frailty Phenotype as a covariate, adjusted for treatment group (1), and for treatment group, age, sex at birth and ASCVD score at enrollment (2). Non-CV deaths without MACE were treated as competing events. Type 3 p-values for the overall effect of frailty status are shown. For visual purposes, hazard ratio (HR) with CI are shown in the log scale.
N denotes number of participants at risk, IR incidence rate of MACE, PY person-years of follow-up, #events number of MACE, HR hazard ratio and CI confidence interval.
